# Study on the Influence of Nano-OvPOSS on the Compatibility, Molecular Structure, and Properties of SBS Modified Asphalt by Molecular Dynamics Simulation

**DOI:** 10.3390/polym14194121

**Published:** 2022-10-01

**Authors:** Lei Feng, Peng Zhao, Tongdan Chen, Minghai Jing

**Affiliations:** School of Materials Science and Engineering, Chang’an University, Xi’an 710061, China

**Keywords:** nano-OvPOSS, SBS, modified asphalt, compatibility, structure, mechanical properties

## Abstract

The present research is carried out to inspect the influence of nano-OvPOSS (octavinyl oligomeric silsesquioxane) with different particle sizes on styrene-butadiene-styrene (SBS) modified asphalt through the method of molecular dynamics simulation. This nanomaterial is investigated for the first time to be used in asphalt modification. With the construction of modified asphalt simulation models and the analysis of their mixing energy, radius of gyration (R_g_), radial distribution function (RDF), ratio of free volume (RFV), heat capacity, bulk modulus, and shear modulus, this study elucidates the influence of nano-OvPOSS on the compatibility between SBS and asphalt, on the structure of SBS as well as that of asphalt molecules and on the temperature stability and mechanical properties of SBS modified asphalt. The results show that nano-OvPOSS not only is compatible with SBS as well as with asphalt, but also is able to improve the compatibility between SBS and asphalt. Nano-OvPOSS is able to reinforce the tractility of branched chains of SBS and make SBS easier to wrap the surrounding asphalt molecules. The free movement space of molecules in the SBS modified asphalt system also shrinks. Moreover, the addition of nano-OvPOSS into SBS modified asphalt results in higher heat capacity, bulk modulus, and shear modulus of modified asphalt. All of these effects contribute to a more stable colloidal structure as well as more desirable temperature stability and deformation resistance of the modified asphalt system. The overall results of the study show that nano-OvPOSS can be used as a viable modifier to better the performance of conventional SBS modified asphalt.

## 1. Introduction

Polymer modifiers are widely used to improve the properties of asphalt to obtain low-temperature crack resistance, high-temperature rutting resistance, and fatigue resistance [1,2,3,4]. Styrene-butadiene-styrene (SBS), a kind of thermoplastic elastomer, is universally employed as an asphalt modifier. SBS modified asphalt is characterized as neither being viscous when heated nor being fragile when cooled, which is of great plasticity and property of aging resistance [5,6,7]. It has been found that the properties of the modified asphalt are closely related to the amount of SBS, considering that it has a close relation with the microstructure of the SBS modified asphalt. With a small amount, SBS disperses evenly in the asphalt in the form of dispersion medium. As the amount increases, cross-linked SBS networks are synthesized, forming the ideal microstructure of polymer-modified asphalt—the interlock network structure—thereby improving the mechanical and rheological properties of asphalt. The amount of SBS added into the asphalt is normally lower than 10% [8,9,10].

Generally, SBS modified asphalt still needs some improvements. Due to the relatively large difference in solubility and density between SBS and asphalt, this type of system is thermodynamically unstable and a phase separation (SBS-rich phase and asphalt-rich phase) is prone to occur under the influence of gravitational fields [11,12]. Meanwhile, its susceptibility to degradation and aging under the influence of sunlight, oxygen, and heat significantly also minimizes its positive effect on asphalt [13,14,15]. To meet all-round requirements of asphalt modifier, the composite modification method is carried out in which nanomaterial is added with polymer to modify asphalt, which not only makes good use of the skeleton structure of nanomaterial, but also gives full play to polymer’s advantages [16].

Various studies conducted previously have provided positive proof that the addition of nanomaterials to SBS modified asphalt is able to better the performance of SBS modified asphalt. Han et al. [17,18,19,20] researched nano-montmorillonite/SBS composites modified asphalt and discovered that the addition of nano-montmorillonite improves the properties of SBS in high or low temperature and effectively prevents the dissociation situation and at the same time refines the ageing resistance of asphalt. Yet, these studies have only investigated macroscopic properties without the exploring modification mechanism from the molecular level. In 2005, Ouyang et al. [21] studied SBS/Kaolin clay composites modified asphalt and found that Kaolin clay can efficaciously adjust the density difference between SBS and asphalt so that the gathering trend of the SBS particles is slowed. However, as is mentioned in their study, Kaolin clay plays an insignificant role in improving the mechanical properties of asphalt. In 2016, Rezaei et al. [22] discovered that the dynamic shear modulus and rutting resistance factor of compound modified asphalt become prominently higher because of the addition of nano-SiO_2_/SBS. However, this research was limited to only studying the rheological properties of the influence of nano-SiO_2_/SBS on asphalt. In 2020, Su et al. [23] used the molecular dynamics method to study nano-ZnO/SBS compound modified asphalt and found that the addition of nano-ZnO ameliorates the property of SBS modified asphalt in high temperature as well as the shear-resistant property of SBS modified asphalt. What is worth mentioning is that these studies all adopted the kind of nanomaterial that is either metal oxide material or non-metal oxide material, which in fact is unfavorably compatible with asphalt and easy to aggregate in the asphalt system. Thus, despite the fact that the performance of modified asphalt is improved, some negative situations such as the less desired compatibility of nanomaterial with asphalt and the aggregation of nanomaterial in asphalt systems are very likely to occur at the same time. 

To offset such deficiency, POSS (polyhedral oligomeric silsesquioxane), a kind of organic-inorganic nanohybrid material possessing both the properties of organic and inorganic materials, was explored as an asphalt modifier in this study in an attempt to avoid the occurrence of the above-mentioned unfavorable situations and at the same time better the performance of modified asphalt on the whole. This kind of nanomaterial was inspected for the first time in the field of asphalt modification.

POSS was first synthesized in 1946 by Scott [24]. Afterwards, many kinds of POSS were prepared and utilized, such as OvPOSS, CpPOSS, and CyPOSS [25,26,27,28,29,30]. Composed of silicon and oxygen and with a cage-like skeleton structure whose surface is covered by organic groups, POSS is easy to functionalize by changing the organic groups, which bolsters up its miscibility with asphalt and allows for extra convenience of its incorporation into polymer through blending. Moreover, after the hybrid reaction between the polymer and POSS, the properties of the oxidation resistance, flame retardancy, and aging resistance of the polymer are consolidated [31,32,33,34].

Nano-OvPOSS (octavinyl oligomeric silsesquioxane), containing a vinyl group that is also a common organic group in SBS, was selected as the study object in this research owing to its similarity in structure with SBS so that the reciprocal effect between nano-OvPOSS and SBS in the modified asphalt system can be smoothed [9,28,31]. The effect of nano-OvPOSS on the compatibility, structure, and properties of SBS modified asphalt was investigated using the molecular dynamics (MD) simulation method presenting corresponding molecular models of asphalt materials on the basis of experimental data. The MD method is based on Newton’s law and predicts the macroscopic performance of materials by calculating the intra- and intermolecular interactions. Properties derived from the simulation algorithm are similar to those of real asphalt. In this study, the structure-property relationship in the organic-inorganic asphalt blends was analyzed by MD simulation which is intended to bridge the microscopic mechanism and macroscopic properties of materials in the field of asphalt modifier research.

In a nutshell, through MD simulation method, a series of modified asphalt models were constructed and the mixing energy, radius of gyration (R_g_), radial distribution function (RDF), ratio of free volume (RFV), the heat capacity, the bulk modulus, and shear modulus were utilized in this research to analyze the compatibility, molecular structure, temperature stability, and mechanical properties of modified asphalt system, with the intention of gaining a brief glimpse of the application of nano-OvPOSS in modified asphalt system.

## 2. Simulation Models

### 2.1. Molecular Model of Matrix Asphalt

This study underwent a separation test to obtain the proportion of the four components of the asphalt simulation model. The PG 64-16 matrix asphalt, which is common in the construction of asphalt pavement in China, was used for the separation test. The physical properties of matrix asphalt are shown in Table 1. According to ASTM D4124 standard, the separation test was carried out, and the four components of asphalt were defined as asphaltene, resin, saturate, and aromatic. The separation test results are listed in Table 2.

**Figure 1:**
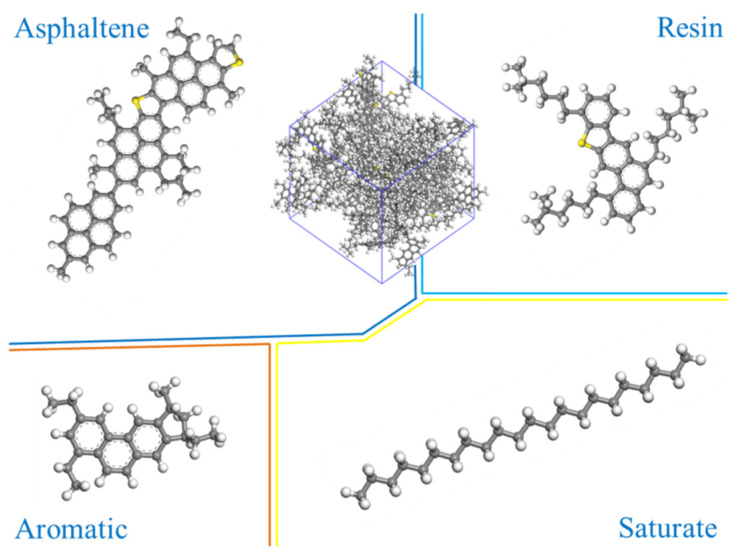
Molecular structure models of four asphalt components and matrix asphalt (carbon atoms are gray, sulfur atoms are yellow, and hydrogen atoms are white).

The four components of asphalt model were constructed with reference to the results conducted by Hansen et al. [35]. From the separation test results of matrix asphalt, the ratio of each molecule in asphalt simulation system was calculated, as is listed in Table 3. The four components of matrix asphalt and the constructed matrix asphalt simulation model are shown in Figure 1.

### 2.2. Molecular Model of Nano-OvPOSS

**Figure 2:**
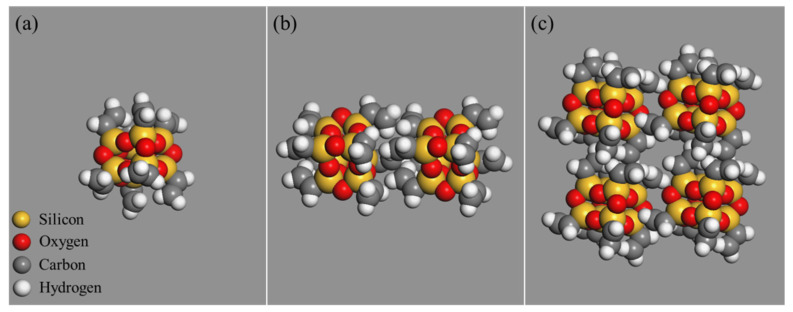
OvPOSS nanoclusters with different sizes: (**a**) 5 Å; (**b**) 7 Å; (**c**) 9 Å (silicon atoms are orange and oxygen atoms are red).

Using the cluster building tool, nanoclusters with three different sphere diameters were built, that are 5 Å, 7 Å, and 9 Å. The sphere diameters of nanoclusters are referred to as the size of nanoclusters in this research. The parameters of OvPOSS are listed in Table 4. The constructed nano-OvPOSS nanoclusters with three kinds of diameters are shown in Figure 2.

### 2.3. Molecular Model of Linear SBS

**Figure 3:**
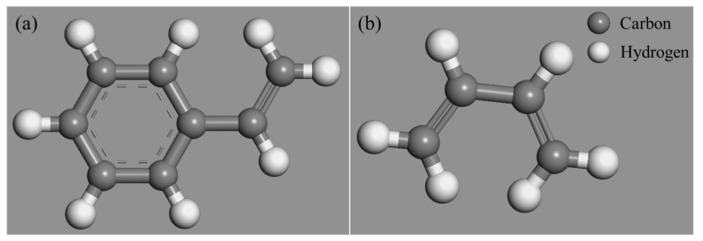
Monomer molecular models: (**a**) styrene; (**b**) 1,3-butadiene.

**Figure 4:**
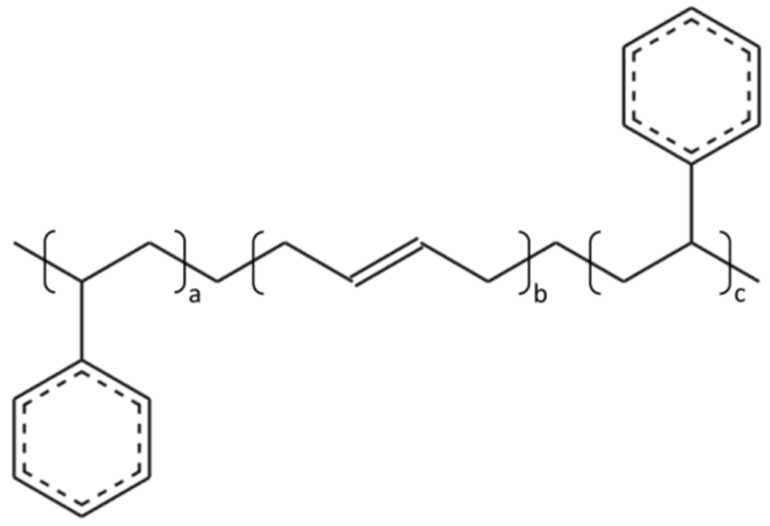
Molecular formula of linear SBS.

**Figure 5:**
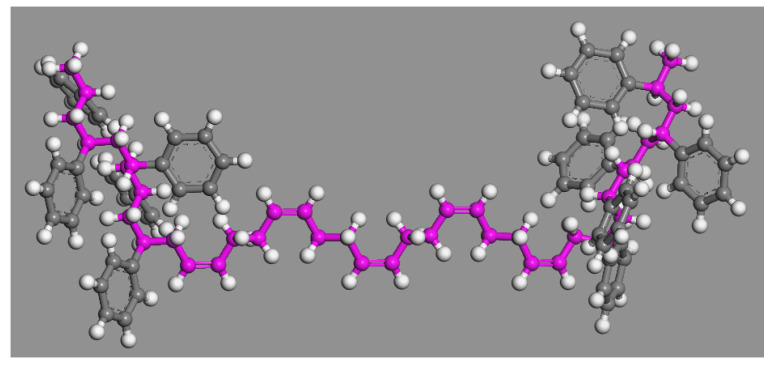
Molecular model of linear SBS.

The linear SBS, derived from styrene and 1,3-butadienem (Figure 3), was used in this research, whose molecular formula and molecular model are shown in Figure 4 and Figure 5.

### 2.4. Molecular Model of Nano-OvPOSS/SBS Modified Asphalt

**Figure 6:**
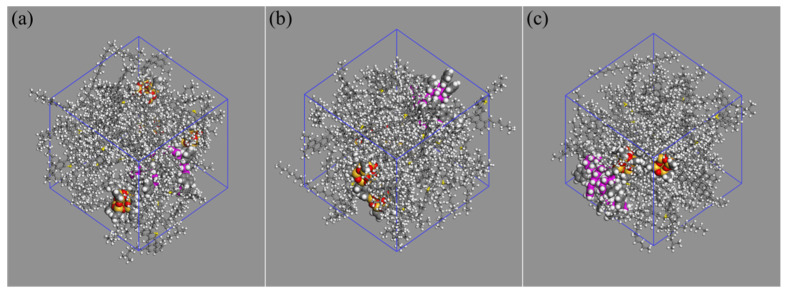
Nano-OvPOSS/SBS modified asphalt models with different nano-OvPOSS diameters: (**a**) 5 Å nano-OvPOSS; (**b**) 7 Å nano-OvPOSS; (**c**) 9 Å nano-OvPOSS.

The nano-OvPOSS/SBS modified asphalt models with different nano-OvPOSS diameters were constructed by Amorphous cell module, as is shown in Figure 6. In most of SBS modified asphalt experiments, the dosage of SBS was often chosen to be around 5% [4,5,6,36]. In this research, the dosage of SBS was chosen to be 4.699%, which was able to ensure the successful building of the OvPOSS/SBS modified asphalt model with one SBS molecule. Through several trials, it was found that if two SBS molecules were added to build the OvPOSS/SBS modified asphalt model, the content of SBS turned out to be 8.975%, which is much more than the commonly used dosage (5%). Meanwhile, the dosage of nano-OvPOSS was chosen to be 9.054% to meet the least amount requirement for the model construction of nano-OvPOSS/SBS modified asphalt. Detailed information is shown in Table 5.

## 3. MD Simulation Theory and Method

### 3.1. Simulation Task

#### 3.1.1. Mixing Energy

According to the Flory–Huggins model, the expression of free energy of mixing of a binary system can be expressed as Equations (1) and (2):

(1) $$\frac{\Delta G}{RT}=\frac{\phi_{b}}{n_{b}}ln\phi_{b}+\frac{\phi_{s}}{n_{s}}ln\phi_{s}+x\phi_{b}\phi_{s}$$

(2) $$x=\frac{E_{mix}}{k_{B}T}$$

where Δ*G* is the free energy of mixing; *R* is the gas constant; *T* is the temperature; *ϕ_i_* is the volume fraction of *i*; *n_i_* is the degree of polymerization of *i*; *E_mix_* is the mixing energy; *x* is the interaction parameter; and *k_B_* is the Boltzmann constant.

In the traditional Flory–Huggins model, each component occupies a lattice site. For a lattice with coordination number Z, the mixing energy is calculated by Equation (3):

(3) $$E_{mix}=\frac{1}{2}Z\left(E_{bs}+E_{sb}-E_{bb}-E_{ss}\right)$$

where *E_ij_* is the binding energy between a unit of component *i* and a unit of component *j*. 

#### 3.1.2. R_g_

R_g_ is used to reflect the changes of the shape of polymer molecular. The expression of R_g_ is shown in Equation (4).

(4) $$R_{g}={\left(\frac{\sum r^{2}m}{\sum m}\right)}^{\frac{1}{2}}$$

where *r* is the distance; *m* is the molecular mass.

#### 3.1.3. RDF

RDF can be calculated by the ratio of regional density to the average density of the system, the expression of RDF is shown in Equation (5).

(5) $$g\left(r\right)=\frac{dN}{\rho 4\pi r^{2}dr}$$

where g(*r*) is the RDF; *N* is the total number of atoms; *ρ* is the density of system; *r* is the distance.

#### 3.1.4. RFV

Free volume is the unoccupied space between molecules. RFV is the parameter to express the percentage of the volume not occupied by molecules, which can be calculated by Equation (6).

(6) $$\mathrm{RFV}=\frac{V_{f}}{V_{f}+V_{o}}\times 100\%$$

where *V_f_* is the free volume; *V_o_* is the occupied volume.

### 3.2. Simulation Method

The asphalt models with three dimensional periodic conditions were built according to related parameters by the Amorphous cell tool. The geometry optimization was employed to optimize the structure of model, and then the dynamic process for 2000 ps was employed to ensure a stable asphalt molecular configuration. To obtain the true global potential energy minimum configuration, the anneal process was used to overcome the migration energy barrier in the modified asphalt system. In this process, the prepared asphalt models were initially heated up to 500 K and then cooled to 298 K, and this anneal process was carried out four times for a total of 2000 ps. After these relaxation procedures, the geometry optimization was conducted for a second time, then the molecular dynamic process with the NPT ensemble for 1000 ps at 298 K was followed. This molecular dynamic process generated various equilibrium models to be used for subsequent studies. The time step is 1.0 fs. The COMPASS force field [37,38] was adopted to describe the atomistic interactions. The temperature and pressure of the molecular dynamic system were controlled by Nose [39] thermostat and Berendsen [40] barostat separately, and the decay constant of temperature control method and pressure control method were both 0.1 ps.

### 3.3. Model Validation

**Figure 7:**
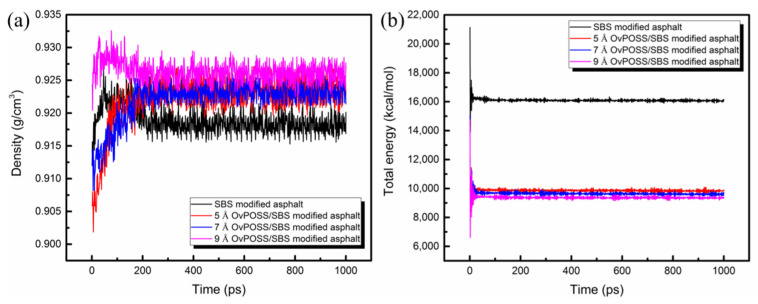
Model validation parameters: (**a**) density; (**b**) energy.

The accuracy of simulation models and methods can be verified through two important indicators—density and energy. Thus, these two indicators of SBS modified asphalt system and nano-OvPOSS/SBS modified asphalt systems were studied. Figure 7 shows the density and the energy of the four asphalt systems as a function of simulation time. The density and the energy of the four systems can be seen in this study to have reached the equilibrium state within 1000 ps. The dynamic results of the last 600 ps (400–1000 ps) were chosen as the data reference for subsequent calculation, during which time the density profile and the energy profile were able to reach a stable state. It can be seen that the separate addition of the three kinds of nano-OvPOSS increased the density of the SBS modified asphalt system and decreased the total energy of the SBS modified asphalt system.

## 4. Results and Discussion

### 4.1. Compatibility Analysis

The eight vinyl groups of nano-OvPOSS endow this nanomaterial with distinctive potential to be compatible with polymer. This can be supported by the molecular dynamics method that simulates the probability distribution of the mixing energy of SBS with nano-OvPOSS of three different particle sizes, the mixing energy of four asphalt components with nano-OvPOSS of three different particle sizes, and the effect of nano-OvPOSS on the mixing energy of SBS with asphalt. The compatibility analysis is based on the Flory–Huggins model [41,42,43,44,45]. Details are as the following.

#### 4.1.1. The Compatibility of Nano-OvPOSS with SBS

**Figure 8:**
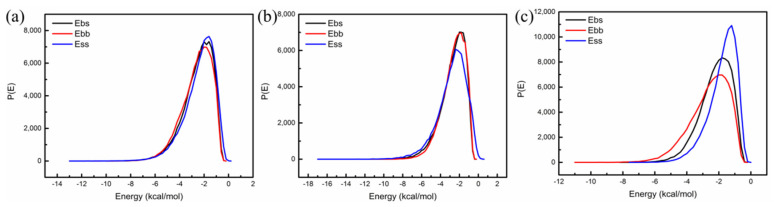
The probability distribution of mixing energy of nano-OvPOSS with SBS: (**a**) 5 Å nano-OvPOSS; (**b**) 7 Å nano-OvPOSS; (**c**) 9 Å nano-OvPOSS.

**Figure 9:**
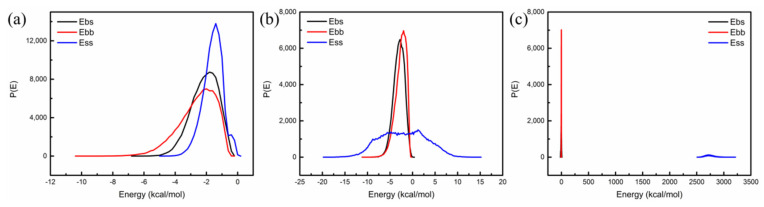
The probability distribution of mixing energy of traditional nanomaterials with SBS: (**a**) SiO_2_; (**b**) ZnO; (**c**) TiO_2_.

Nano-OvPOSS was used as the screen and SBS as the base. The probability distributions of mixing energy of SBS with nano-OvPOSS (5 Å, 7 Å, and 9 Å) were calculated by using Blends tool, as is shown in Figure 8. For further clarification, study of the mixing energy of SBS with traditional nanomaterials of SiO_2_, ZnO, and TiO_2_ (4.4 Å) was cited in this part to facilitate the comparison, which is shown in Figure 9. 

It can be seen that the curves of E_bb_ (base–base), E_bs_ (base–screen), and E_ss_ (screen–screen) of SBS with nano-OvPOSS (5 Å, 7 Å, and 9 Å) in Figure 8 are very similar to each other. In contrast, the curves of that of SBS with SiO_2_, ZnO, and TiO_2_ (4.4 Å) in the three respective pictures in Figure 9 are obviously different from each other. Generally, the similarity of the curves of E_bb_, E_bs_, and E_ss_ reflects the degree of the compatibility of two materials. It is usually accepted that the more similar the probability distributions of E_bb_, E_bs_, and E_ss_ of two materials are, the better the compatibility of the two materials is. Therefore, the above simulation results show that nano-OvPOSS has desirable compatibility with SBS while SiO_2_, ZnO, and TiO_2_ do not. Obviously, nano-OvPOSS, which is organic-inorganic, outperforms traditional nanomaterials SiO_2_, ZnO, and TiO_2_, which are metal oxide or non-metal oxide, in its compatibility with SBS. This demonstration of poor compatibility of SBS with SiO_2_, ZnO, and TiO_2_ is ascribed to their total difference from polymer (SBS) in the structure and composition, while nano-OvPOSS is endowed with desired performance of compatibility thanks to its similar structure and composition with polymer (SBS) in accordance with the similar dissolve mutually theory.

#### 4.1.2. The Compatibility of Nano-OvPOSS with Four Asphalt Components

**Figure 10:**
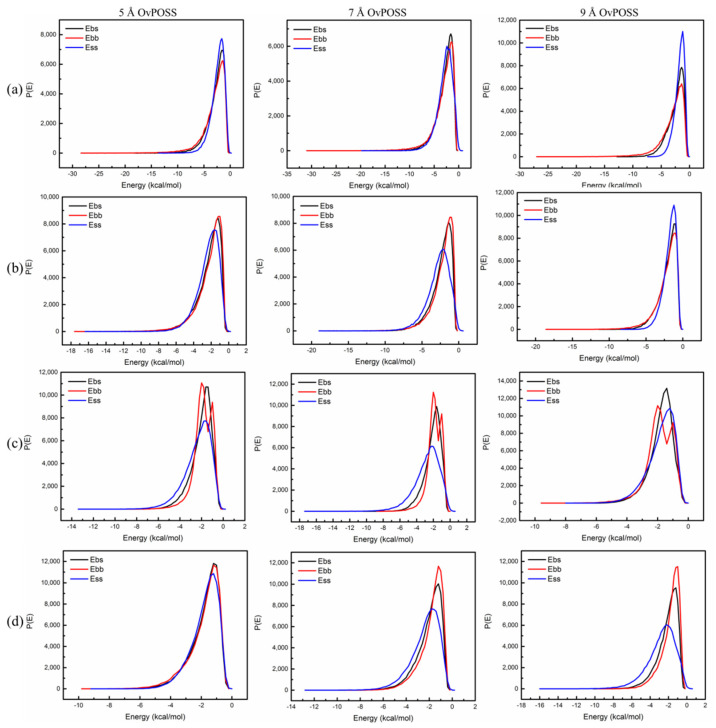
The probability distribution of mixing energy of nano-OvPOSS (5 Å OvPOSS, 7 Å OvPOSS, and 9 Å OvPOSS) with four asphalt components: (**a**) asphaltene; (**b**) resin; (**c**) saturate; (**d**) aromatic.

Nano-OvPOSS was used as the screen, and four asphalt components as the base. The probability distributions of mixing energy of nano-OvPOSS (5 Å, 7 Å, and 9 Å) with the four asphalt components were calculated as is shown in Figure 10. Figure 10a represents the probability distribution of mixing energy of asphaltene with 5 Å nano-OvPOSS, 7 Å nano-OvPOSS, and 9 Å nano-OvPOSS; subsequently, Figure 10b–d show the probability distribution of mixing energy of resin with 5 Å nano-OvPOSS, 7 Å nano-OvPOSS, and 9 Å nano-OvPOSS, the probability distribution of mixing energy of saturate with 5 Å nano-OvPOSS, 7 Å nano-OvPOSS, and 9 Å nano-OvPOSS, and the probability distribution of mixing energy of aromatic with 5 Å nano-OvPOSS, 7 Å nano-OvPOSS, and 9 Å nano-OvPOSS, respectively. 

In comparison with Figure 9, each of the twelve pictures of Figure 10 shows obvious similarity of E_bb_, E_bs_, and E_ss_ with each other, which means 5 Å nano-OvPOSS, 7 Å nano-OvPOSS, and 9 Å nano-OvPOSS are favorably compatible with the four components of asphalt. 

As is shown in the four pictures of 5 Å nano-OvPOSS with asphaltene, resin, saturate, and aromatic, the three curves of E_bb_, E_bs_, and E_ss_ in each picture express the most favorable similarity with each other, which are compared with the respective four pictures of 7 Å nano-OvPOSS with asphaltene, resin, saturate, and aromatic as well as with the respective four pictures of 9 Å nano-OvPOSS with asphaltene, resin, saturate, and aromatic. It can be concluded that 5 Å nano-OvPOSS has the most favorable compatibility with asphaltene, resin, saturate, and aromatic when compared with 7 Å nano-OvPOSS and 9 Å nano-OvPOSS.

Meanwhile, Figure 10a,d express clear variation from similarity to difference of the three curves of E_bb_, E_bs_, and E_ss_ with the particle size of nano-OvPOSS increasing from 5 Å to 9 Å. Conclusions can be drawn that the compatibility between nano-OvPOSS and asphaltene as well as the compatibility between nano-OvPOSS and aromatic are influenced to a large extent by the particle size of nano-OvPOSS. The smaller the particle size of nano-OvPOSS is, the better the compatibility with asphaltene and aromatic is.

#### 4.1.3. The Effect of Nano-OvPOSS on the Compatibility of SBS Modified Asphalt

**Figure 11:**
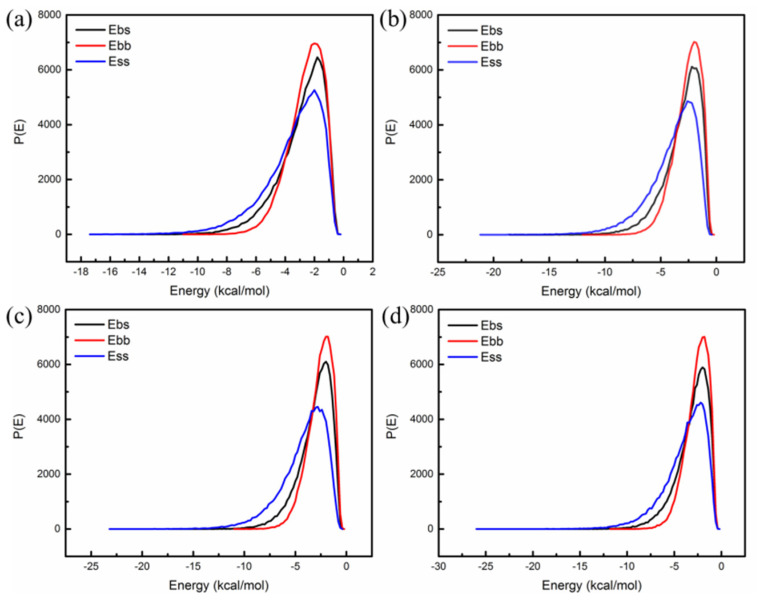
The probability distribution of mixing energy of SBS with asphalt blends: (**a**) matrix asphalt/SBS; (**b**) 5 Å nano-OvPOSS/asphalt/SBS; (**c**) 7 Å nano-OvPOSS/asphalt/SBS; (**d**) 9 Å nano-OvPOSS/asphalt/SBS.

The asphalt blends were used as the screen and SBS as the base. Figure 11a shows the probability distribution of mixing energy of matrix asphalt/SBS without nano-OvPOSS; Figure 11b–d show the probability distribution of mixing energy of 5 Å nano-OvPOSS/asphalt/SBS, 7 Å nano-OvPOSS/asphalt/SBS, and 9 Å nano-OvPOSS/asphalt/SBS, respectively. As can be seen from Figure 11a, the probability distribution curves of E_bb_, E_bs_, and E_ss_ of matrix asphalt/SBS are very similar to each other, which signifies that SBS has good compatibility with asphalt. The similarity of the curves of E_bb_, E_bs_, and E_ss_ of 5 Å nano-OvPOSS/asphalt/SBS can also be found in Figure 11b. Such similarity indicates the compatibility between SBS and asphalt are not changed by the addition of nano-OvPOSS and 5 Å nano-OvPOSS/asphalt/SBS are compatible with each other. The same conclusion can also be drawn from Figure 11c that 7 Å nano-OvPOSS/asphalt/SBS are compatible with each other and from Figure 11d that 9 Å nano-OvPOSS/asphalt/SBS are compatible with each other.

**Figure 12:**
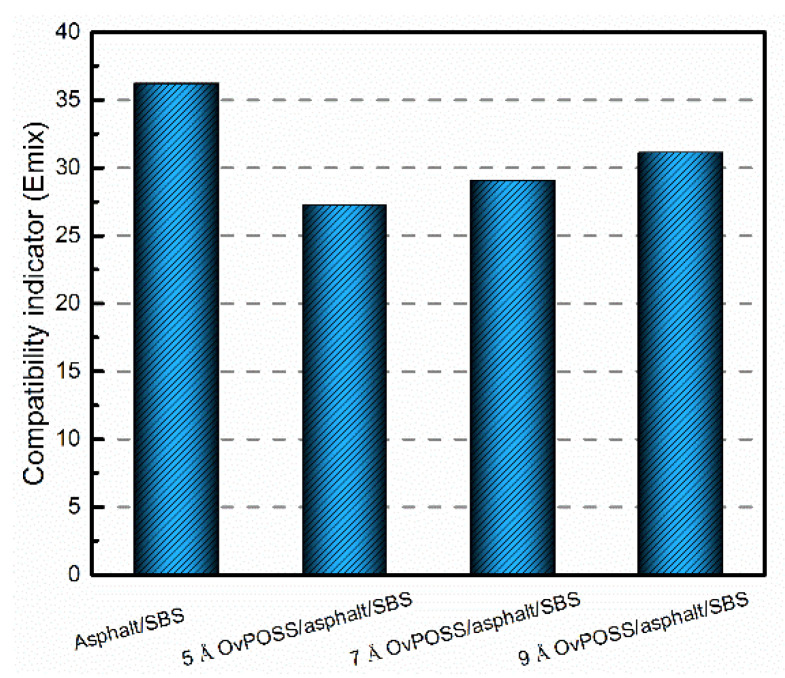
Compatibility indicator E_mix_ of modified asphalt.

The quantitative analysis of the effect of nano-OvPOSS on the compatibility of SBS modified asphalt can be taken from Figure 12 which expresses the compatibility indicator E_mix_ of modified asphalt. If the absolute value of E_mix_ is the smallest, the compatibility of two materials is the best. It can be seen from Figure 12 that the absolute value of E_mix_ of 5 Å nano-OvPOSS/asphalt/SBS is the lowest and that of SBS/asphalt is the highest. Moreover, as the particle size of nano-OvPOSS becomes larger, the absolute values of E_mix_ of (5 Å, 7 Å, and 9 Å) nano-OvPOSS/SBS/asphalt increase sequentially. The results show that nano-OvPOSS is able to improve the compatibility between SBS and asphalt, thus easing off the phase separation and guaranteeing SBS modified asphalt a more stable state. Moreover, 5 Å nano-OvPOSS displays the best performance in improving the compatibility between SBS and asphalt.

The eight ethylene organic branches of nano-OvPOSS are capable of interacting with the active groups of polymers, which is why nano-OvPOSS is able to improve the compatibility between SBS and asphalt. It is well accepted that the outstanding performance of nanomaterial, such as their large specific surface area and high strain resistance, attributes to their unique small size [46,47,48]. It is also accepted that the smaller the size of nanomaterial, the larger their specific surface area will be. Therefore, the smallest particle size of nano-OvPOSS in this study, 5 Å nano-OvPOSS, has the largest contact range with polymer molecules, which gives it the best compatibility between SBS and asphalt compared with 7 Å nano-OvPOSS and 9 Å nano-OvPOSS. 

The validation of the favorable compatibility between the organically processed nanomaterials and asphalt has actually been confirmed previously by several related experiments. In 2020, Fanourakis et al. [49] found that the solubility of the organically treated oxide MoO_3_ in organic matter was improved, and its aggregation behavior was significantly reduced. In 2020, Zhang et al. [50,51] used a high-temperature storage stability test to estimate the compatibility between nanomaterial and asphalt. They found that after organic modification, the ΔS were further reduced to 0.5 °C in ODBA-REC (octadecyl dimethyl benzyl ammonium chloride-rectorite) modified asphalt since the benzyl chain in ODBA increases the adsorption effect of asphalt molecules on individual REC layers, thereby contributing to the desired compatibility between the ODBA-REC and asphalt. In this study, that nano-OvPOSS is viable in improving the compatibility with asphalt not only can be confirmed by the MD studies stated and analyzed above, but also can be drawn from its structural advantages. Basically, the structural equivalence guarantees the compatibility of different materials. Nano-OvPOSS is capable of displaying desired compatibility with SBS as well as with asphalt because it possesses both the properties of organic material and those of inorganic material as an organic-inorganic nano-hybrid material. Furthermore, it can improve the compatibility between SBS and asphalt in the meantime. Consequently, nano-OvPOSS outperforms the traditionally used nanomaterials such as SiO_2_, ZnO, and TiO_2_ as an ideal asphalt modifier when taking the promotion of the compatibility with polymer into account.

### 4.2. Influence of Nano-OvPOSS on the Structure of SBS Modified Asphalt

#### 4.2.1. Influence of Nano-OvPOSS on R_g_ of SBS

**Figure 13:**
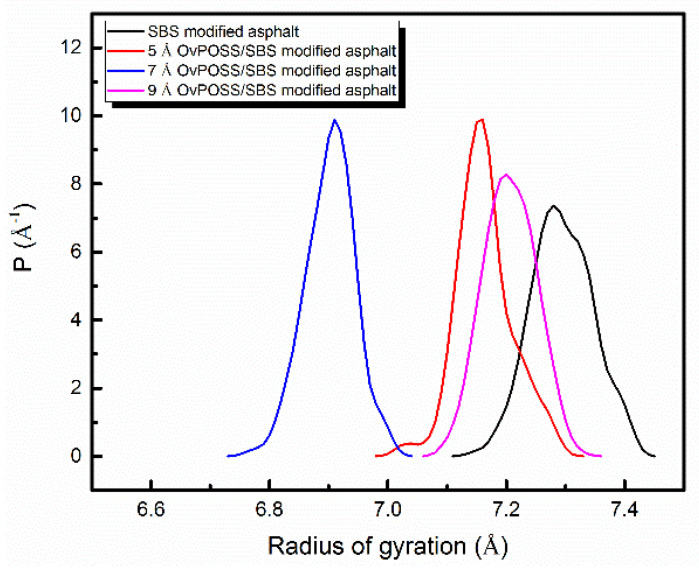
R_g_ of SBS in different modified asphalt systems.

**Figure 14:**
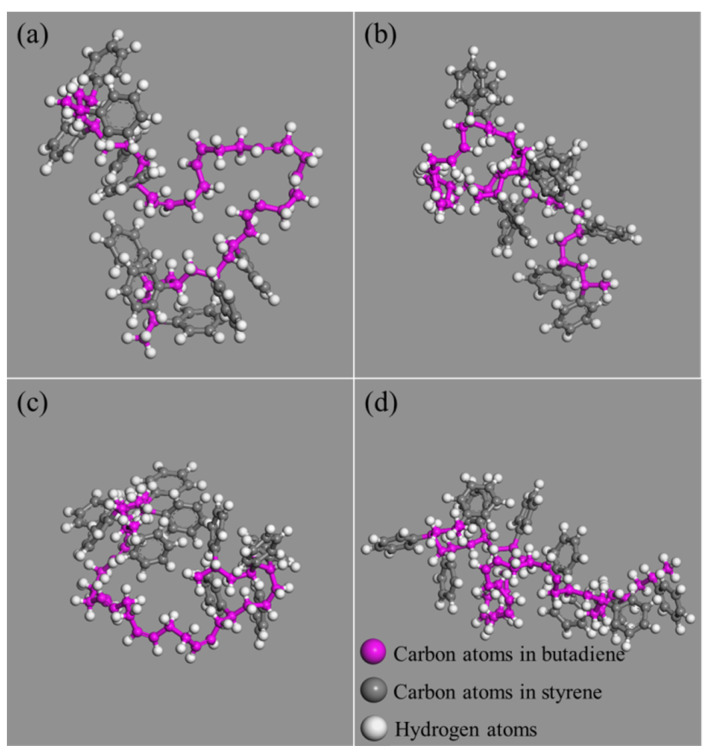
Structure of SBS in different modified asphalt systems: (**a**) SBS modified asphalt; (**b**) 5 Å nano-OvPOSS/SBS modified asphalt; (**c**) 7 Å nano-OvPOSS/SBS modified asphalt; (**d**) 9 Å nano-OvPOSS/SBS modified asphalt.

R_g_ is often used to characterize the dynamic trajectory of flexible systems for molecular systems. A small R_g_ indicates the polymer is relatively compact, meaning throughout its trajectory the polymer spends most of its time as a folded structure. Figure 13 and Figure 14 present respectively the changes of R_g_ and structures of SBS before and after the addition of nano-OvPOSS with different particle sizes into SBS modified asphalt. As is shown, without nano-OvPOSS, the R_g_ peak position of SBS is 7.28 Å and the width of R_g_ peak is 0.33 Å. With the separate addition of nano-OvPOSS with three different particle sizes, the R_g_ peak position of SBS shifts to 7.15 Å, 6.91 Å, and 7.20 Å. It reveals that the addition of nano-OvPOSS leads to the collapse of SBS molecule and increases the compactness of SBS. It is also notable that the 7 Å nano-OvPOSS has a more discernible effect on the compactness of SBS. 

On the one hand, the SBS chain collapses in a crowded environment after the addition of nano-OvPOSS; on the other hand, the different shapes of the crowded particles bring out the different structures of SBS after the collapse [52], as is shown in Figure 14. Notably, the R_g_ peak width of SBS is narrowed to 0.31 Å, 0.26 Å, and 0.29 Å, respectively. The decrease of the peak width indicates that SBS branched chain reinforces its tractility and becomes easier to wrap the surrounding asphalt molecules after the addition of nano-OvPOSS, which promotes its attraction of the surrounding asphalt molecular [23].

#### 4.2.2. Influence of Nano-OvPOSS on RDF of SBS Modified Asphalt

**Figure 15:**
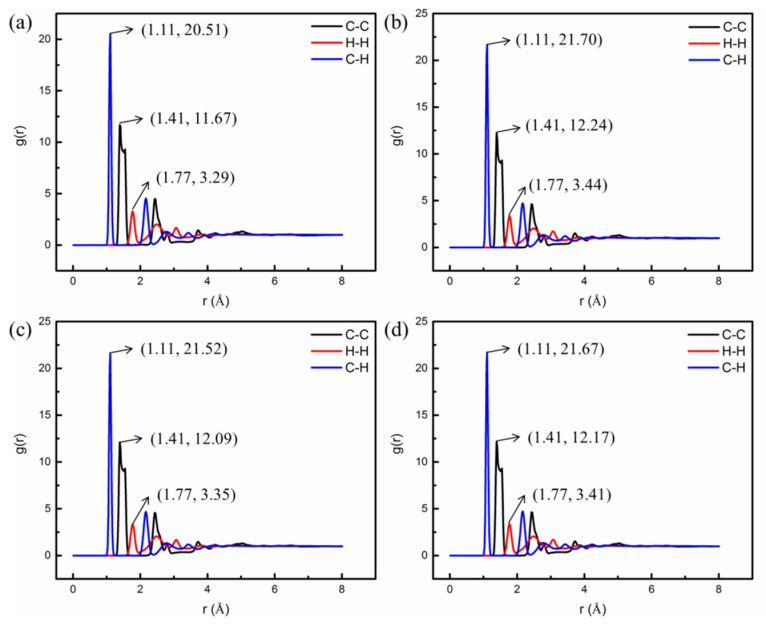
RDF of atoms in four modified asphalt systems: (**a**) SBS modified asphalt; (**b**) 5 Å OvPOSS/SBS modified asphalt; (**c**) 7 Å OvPOSS/SBS modified asphalt; (**d**) 9 Å OvPOSS/SBS modified asphalt.

The RDF is a measure of the relative probability of finding a particle at a distance r from a reference particle, and it mainly characterizes the packing state of atoms and the distance between atoms. To study the influence of nano-OvPOSS on RDF of atoms in SBS modified asphalt system, a carbon atom and hydrogen atom were selected as reference and selection for research. Figure 15 shows the RDF of atoms in a SBS modified asphalt system before and after the addition of nano-OvPOSS (5 Å, 7 Å, and 9 Å).

According to Figure 15, before the addition of nano-OvPOSS, the curve of RDF has the first peak at 1.11 Å, representing the distance between carbon atom and hydrogen atom in the system; the second peak of the curve of RDF is at 1.41 Å, representing the distance between carbon atoms in the system; the third peak of the curve of RDF is at 1.77 Å, representing the distance between hydrogen atoms in the system. After adding 5 Å, 7 Å, and 9 Å nano-OvPOSS separately, the positions of the first peak, second peak, and third peak of the curve of RDF remain unchanged, which means the addition of nano-OvPOSS has no influence on the chemical structure of SBS modified asphalt system. On the other hand, before the addition of nano-OvPOSS, the intensity of the first peak, second peak, and third peak of SBS modified asphalt system is 20.51%, 11.67%, and 3.29%, respectively. With the addition of 5 Å nano-OvPOSS, the intensity increases to 21.70%, 12.24%, and 3.44% respectively; with the addition of 7 Å nano-OvPOSS, the intensity increases to 21.52%, 12.09%, and 3.35%; with the addition of 9 Å nano-OvPOSS, the intensity increases to 21.67%, 12.17%, and 3.41%. These increments suggest that the atomic packing density of C-H, C-C, and H-H is enhanced because of the addition of nano-OvPOSS. Among them, 5 Å nano-OvPOSS results in the most prominent effect in enhancing the atomic packing density. Such strengthened interaction between atoms in asphalt molecules is basically derived from the interaction of the eight vinyl groups of nano-OvPOSS with asphalt molecules.

#### 4.2.3. Influence of Nano-OvPOSS on RFV of SBS Modified Asphalt

**Figure 16:**
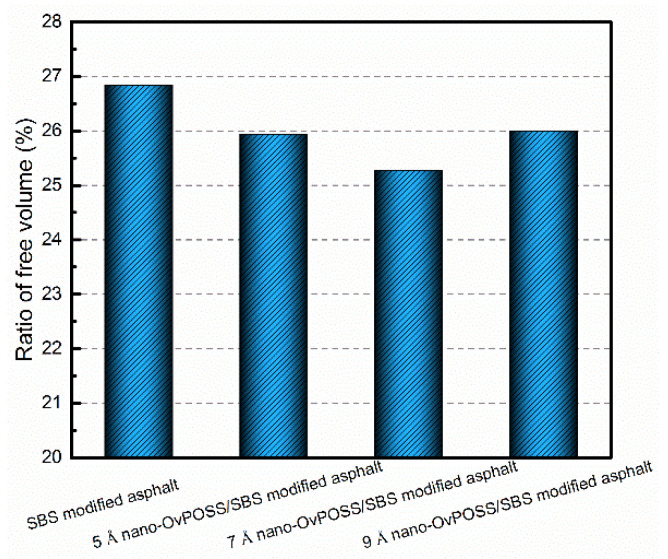
RFV of modified asphalt systems.

Free volume theory has been applied to predict the variation of viscosity and diffusivity of polymeric materials [53,54]. If the free volume fraction of asphalt reduces, its fluidity declines and thus its viscosity becomes higher, which in turn fortifies the deformation resistance of asphalt. Figure 16 describes the RFV of modified asphalt system in which the value of nano-OvPOSS/SBS modified asphalt of different nano-OvPOSS particle sizes (5 Å, 7 Å, and 9 Å) decreases by 3.39%, 5.85%, and 3.13% after the addition of nano-OvPOSS. This indicates that the free movement space of molecules in the modified asphalt system shrinks and the fluidity is hindered, so the addition of nano-OvPOSS increases the viscosity and enhances the deformation resistance of asphalt. This beneficial influence of nano-OvPOSS also derives from its unique structure of eight vinyl groups as an organic-inorganic nanohybrid material, because of which nano-OvPOSS reinforces its combination with molecules in asphalt system, boosts the non-bonding interactions between molecules, and then constructs a stable structural asphalt system. Notably, the 7 Å nano-OvPOSS performs better in reducing the RFV than 5 Å nano-OvPOSS and 9 Å nano-OvPOSS.

### 4.3. The Effect of Nano-OvPOSS on the Temperature Stability of SBS Modified Asphalt 

**Figure 17:**
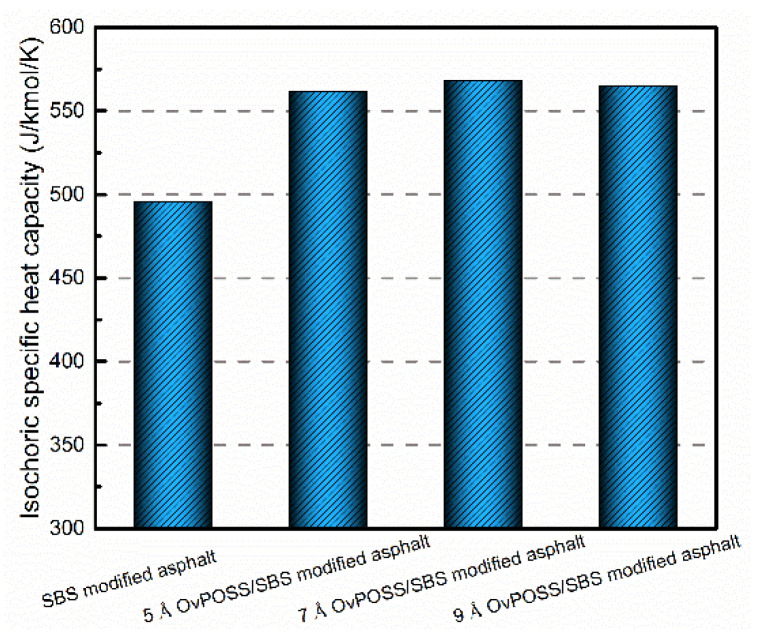
The isochoric specific heat capacity of modified asphalt.

Heat capacity is defined as the amount of heat energy required to raise the temperature of a given quantity of matter by one degree Celsius. The larger the heat capacity, the more heat it absorbs. Therefore, the laws of thermodynamics are used to design new processes for reactions that would have high efficiency. In this study, the isochoric specific heat capacity (C_v_) of modified asphalt systems was simulated at room temperature. The simulation results of C_v_ are shown in Figure 17. It can be seen that the results of the C_v_ of the three nano-OvPOSS/SBS modified asphalt systems are basically the same (because of the same addition content of nano-OvPOSS), and all are obviously higher than that of SBS modified asphalt. This simulation result shows that the nano-OvPOSS improves the C_v_ of SBS modified asphalt, which suggests nano-OvPOSS/SBS modified asphalt system has better temperature stability. That is to say, the addition of nano-OvPOSS is able to alleviate the thermodynamic instability of SBS modified asphalt, which is mainly because the inorganic body of nano-OvPOSS is capable of absorbing more heat.

### 4.4. The Effect of Nano-OvPOSS on the Mechanical Properties of SBS Modified Asphalt

**Figure 18:**
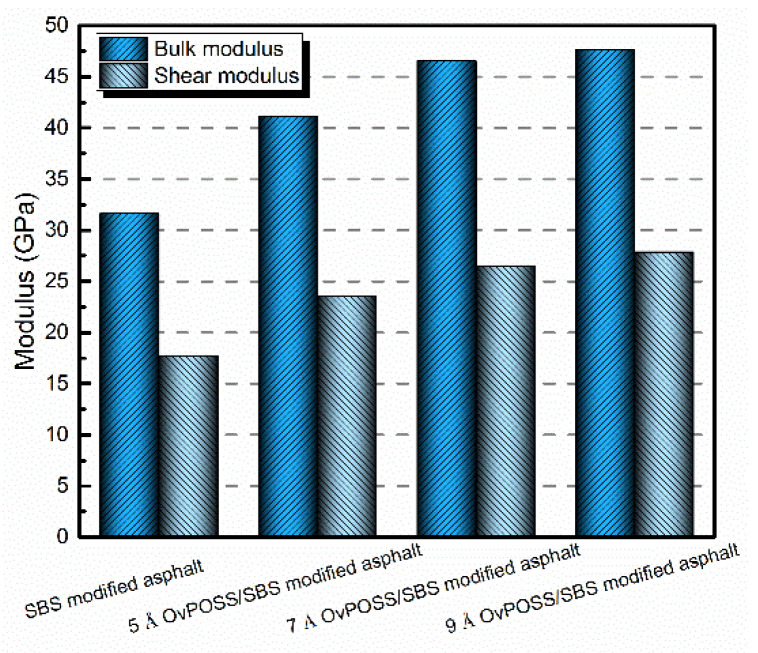
Physical modulus of different modified asphalt systems.

The bulk modulus and shear modulus were simulated to investigate the effect of nano-OvPOSS on the mechanical properties of SBS modified asphalt. The results are shown in Figure 18. As can be seen, compared with SBS modified asphalt, the bulk modulus of nano-OvPOSS/SBS modified asphalt with different nano-OvPOSS particle size (5 Å, 7 Å, and 9 Å) increases by 30.15%, 47.11%, and 50.57%, respectively, and the shear modulus of these asphalt blends increases by 32.58%, 49.15%, and 57.11%, respectively. Thus, nano-OvPOSS has a positive effect on the two moduli of SBS modified asphalt. Such increment helps to equip asphalt with better mechanical properties and exceptional ability to resist deformation, thereby bringing out the desired performance in asphalt pavement. These simulation results are in agreement with laboratory test results [36]. In addition, the maximum values of bulk modulus and shear modulus of nano-OvPOSS/SBS modified asphalt are both obtained when the particle size of nano-OvPOSS is 9 Å. Therefore, the combination of 9 Å nano-OvPOSS and SBS in modifying asphalt is able to acquire the best deformation resistance ability of asphalt.

## 5. Conclusions

In this research, the influence of nano-OvPOSS with different particle sizes on SBS modified asphalt was inspected through the MD method. Conclusions are the following.

- Nano-OvPOSS is compatible with SBS as well as with four asphalt components, and is also able to improve the compatibility between SBS and asphalt. This is because the eight ethylene organic branches of nano-OvPOSS are capable of interacting with the active groups of polymers. Furthermore, 5 Å nano-OvPOSS has better ability to improve the compatibility between SBS and asphalt than 7 Å nano-OvPOSS and 9 Å nano-OvPOSS since it has the larger contact range with polymer molecules.
- The addition of nano-OvPOSS enhances the inter atomic bonding strength of asphalt component molecules. Meanwhile, it can reinforce the tractility of branched chains of SBS and make SBS easier to wrap and adsorb the surrounding asphalt molecules. Moreover, the free movement space of molecules in the SBS modified asphalt system declines. All of these effects result in a more stable colloidal structure of asphalt.
- The improvement of the C_v_ of SBS modified asphalt suggests that nano-OvPOSS/SBS modified asphalt system has better temperature stability. The reason why the addition of nano-OvPOSS is able to alleviate the thermodynamic instability of SBS modified asphalt is that the inorganic body of nano-OvPOSS is capable of absorbing more heat.
- The nano-OvPOSS modifier has a positive effect on bulk modulus and shear modulus of SBS modified asphalt, which renders better mechanical properties and deformation resistance of SBS modified asphalt in asphalt pavement.
- The overall results of the study prove the feasibility of nano-OvPOSS as an ideal asphalt modifier to attain a well-rounded performance of conventional SBS modified asphalt. As a functional material, POSS is capable of changing its eight substituents to realize more functions, one of which is nano-OvPOSS. Hence, the complexity and diversity of the structures of POSS allow for quite a few possibilities of its application in the field of modified asphalt. It is earnestly anticipated that more and more modifiers taking the advantage of POSS can be explicitly explored by many a researcher in the near future.

## Figures and Tables

**Table 1:** Physical properties of PG64-16 matrix asphalt.

Properties	Test Results	Test Standard
Penetration (0.1 mm) (25 °C, 100 g, 5 s)	73.3	ASTM D5-06
Softening point (°C)	47.5	ASTM D36-06
Ductility (cm) (5 cm/min, 10 °C)	67.2	ASTM D113-07

**Table 2:** Separation test results of PG64-16 matrix asphalt.

Parameter	Asphaltene	Resin	Saturate	Aromatic
Weight (g)	0.074	0.278	0.222	0.386
Percent (%)	7.71	28.96	23.13	40.20

**Table 3:** The information about molecular models of four asphalt components.

Name	Chemical Formula	Number of Molecules	Number of Atoms	Content (%)
Asphaltene	C_64_H_52_S_2_	2	236	7.34
Resin	C_41_H_54_S	12	1152	28.81
Saturate	C_22_H_46_	18	1224	23.17
Aromatic	C_24_H_28_	31	1612	40.68

**Table 4:** The parameters of OvPOSS.

Parameter	Results
Molecular formula	C_16_H_24_O_12_Si_8_
Molecular weight	633
Density (g/cm^3^)	1.22
Melting point (°C)	>350
Flash point (°C)	148.4
Toxicity	Non-toxic

**Table 5:** Detailed information of modified nano-OvPOSS/SBS asphalt system.

Name	Chemical Formula	Content (%)
Asphaltene	C_64_H_52_S_2_	6.331
Resin	C_41_H_54_S	24.842
Saturate	C_22_H_46_	19.992
Aromatic	C_24_H_28_	35.082
OvPOSS	C_16_H_24_O_12_Si_8_	9.054
SBS	C_100_H_112_	4.699
